# A randomized pilot study on the effect of niacin on pulmonary arterial pressure

**DOI:** 10.1186/s13063-015-1013-6

**Published:** 2015-11-21

**Authors:** Martin J. McNamara, Jason J. Sayanlar, Daniel J. Dooley, Monvadi B. Srichai, Allen J. Taylor

**Affiliations:** Cardiology Division, Medstar Heart and Vascular Institute at Medstar Georgetown University Hospital and Medstar Washington Hospital Center, 3800 Reservoir Rd., NW, 5 PHC, Washington, DC 20007 USA

## Abstract

**Background:**

Niacin induces the release of vasodilating prostaglandins, for which receptors are present within the pulmonary arterial circulation. We hypothesized that immediate-release niacin would reduce right ventricular systolic pressure in patients with pulmonary hypertension in a randomized, double-blinded, single-dose provocation study.

**Methods:**

We recruited inpatient subjects with a Doppler echocardiogram showing a peak tricuspid regurgitation (TR) jet velocity of 2.7 m/s or greater, and who were free of known pulmonary vascular disease. Subjects were randomized in a 1:2:2 ratio to receive a single dose of either placebo, niacin 100 mg or niacin 500 mg, respectively. TR jet velocities were measured immediately before, and 1 hour post dose, corresponding to peak niacin absorption and prostaglandin release. The primary endpoint was the change in mean TR jet velocity measured over ten successive cardiac cycles.

**Results:**

The baseline mean estimated right ventricular systolic pressure (RVSP) for all 49 subjects (25 male) was 51.9 ± 12.1 mm Hg. The primary endpoint of mean change in TR jet velocity was 0.016 ± 0.065 m/s in the placebo group, compared to −0.017 ± 0.065 m/s with niacin 100 mg, and −0.063 ± 0.038 m/s with niacin 500 mg (*P* = 0.63). The change in maximum estimated RVSP across the three drug groups was 0.2 ± 1.6 mm Hg, −1.3 ± 1.8 mm Hg and −2.2 ± 1.2 mm Hg (*P* = 0.62). In exploratory pairwise analysis in the high-dose niacin group (500 mg), the reduction in mean RVSP was from 50.9 ± 9.4 mm Hg to 48.7 ± 10.0 mm Hg (*P* = 0.09).

**Conclusions:**

A single dose of immediate-release niacin (100 mg or 500 mg) had no significant effect on RVSP 1 hour post administration. A nonsignificant dose-dependent trend for a modest reduction in RVSP, most notable in the 500 mg group, was noted.

(ISRCTN number 12353191, registered April 23, 2015).

**Electronic supplementary material:**

The online version of this article (doi:10.1186/s13063-015-1013-6) contains supplementary material, which is available to authorized users.

## Background

Pulmonary arterial hypertension (PAH) is a rare but increasingly prevalent disease that is characterized by abnormal proliferation, remodeling, and contraction of endothelial cells and smooth muscle cells within the pulmonary artery in association with increased pulmonary artery systolic pressure [1–3]. All approved treatments used to treat PAH target an observable imbalance of vascular tone through vasoconstricting (thromboxanes) and vasodilating (prostaglandins) mediators [2, 4]. The potent vasoconstrictor thromboxane A_2_ is countered by the vasodilating prostacyclin (PGI_2_). In patients with PAH, thromboxane A_2_ is elevated while prostacyclin is diminished, leading to the high pulmonary pressures and resistance.

Intravenous infusion of prostacyclin was the first drug demonstrated to reduce pulmonary artery systolic pressure and improve survival in patients with PAH [5]. Similarly, other vasodilators shown to be active in the pulmonary arteries include calcium channel blockers, endothelial receptor antagonists, nitric oxide, phosphodiesterase (type 5) inhibitors, and synthetic prostacyclin analogues. However, these treatment options are very costly, difficult to administer, and accompanied by many toxicities and complications [2, 6]. Niacin induces the release of vasodilating prostaglandins, specifically leading to a several hundredfold increase in the concentration of prostaglandin D2 (PGD_2_) [7]. Through activation of the prostaglandin D2 receptor, PGD_2_ mediates the common side effect of cutaneous flushing seen during oral administration. PGD_2_’s G protein-coupled receptor, DP_1_, is also located on vascular muscle cells [8]. Thus, given PAH’s vasoconstricting properties and niacin’s vasodilating abilities, we hypothesized that administration of immediate-release niacin would reduce pulmonary arterial pressure.

## Methods

We conducted a randomized, placebo-controlled, double-blinded, single-center pilot study on the effect of niacin on pulmonary artery pressure. The study was ethically approved by the Georgetown University Hospital Institutional Review Board (GUH IRB) on October 5, 2011 (study number 2012–067). Taking part in the study was entirely voluntary and informed consent was obtained from every participant. A CONSORT flow diagram and checklist are provided at the end of the manuscript under the Additional files section (Additional files 1 and 2). Echocardiographic tricuspid regurgitation (TR) jet velocity was used as an accurate noninvasive surrogate of pulmonary artery pressure [9–11]. Participants were recruited by screening inpatient echocardiogram logs at MedStar Georgetown University Hospital (MGUH) for Doppler TR jet velocity greater than 2.7 meters/second (m/s). Patients excluded from the study include the following: inability to provide written, informed consent, known pulmonary vascular disease, known intolerance to niacin or current treatment with niacin, current treatment with a nonsteroidal anti-inflammatory drug (NSAID), known liver disease (aspartate transaminase/alanine transaminase (AST/ALT) >3 times the upper limit of normal), or patients currently on ventilator support or using a bi-level positive airway pressure (BiPAP) device.

### Study drug

This was a single-dose provocation study evaluating niacin (100 mg or 500 mg) versus placebo, in a 2:2:1 ratio. Niacin was administered in the immediate-release, crystalline form of nicotinic acid. The study drug was provided by a research pharmacy with allocation concealed through administration of niacin within identical capsules that either contained placebo or niacin. The randomization sequence was determined using a random number generator and allocation concealed. Subjects were in the nonfasting state prior to study drug administration, but nothing by mouth (NPO) throughout the study.

### Echocardiography

Doppler echocardiography (Philips IE33, Philips Electronics N.V., Amsterdam, The Netherlands) using a 3.5 mHz transducer was used to measure TR jet velocity immediately pre/post administration of the study drug. Inferior vena cava (IVC) diameter and collapsibility was assessed for estimation of right atrial pressure. After baseline measurements were performed, the patient was immediately administered a single dose of the study medication. Sixty minutes later, corresponding to the usual time of maximal niacin absorption and peak niacin-induced cutaneous flushing, a second echocardiogram was done to reassess TR jet velocity and IVC diameter. To eliminate sonographer variability, the same sonographer performed both the pre and post images. Echocardiography was performed with the patient in the supine position. Doppler recordings of TR jet velocity were acquired using apical, lower left parasternal, and subcostal transducer positions. The best view for TR jet velocity was determined in the baseline echocardiogram. This optimal view of TR jet velocity was used for both the pre and post imaging. The TR jet velocity measurements were determined from the average of ten consecutive cardiac cycles when possible. Otherwise, multiple frames of peaks were captured and the most accurate envelopes were chosen for measurement. TR jet velocities were measured by two independent interpreters unaware of study drug assignment.

### Endpoints and statistical analysis

The primary endpoint was the change in mean TR jet velocity measured in meters/second (m/s). The secondary endpoint was the change in maximum TR jet velocity. Between-group differences in baseline and endpoint variables were performed using analysis of variance (ANOVA) between the three randomization groups. Dose comparisons were adjusted for multiple endpoint testing using a Bonferroni correction. Right ventricular systolic pressure (RVSP) was estimated via the modified Bernoulli equation. A *P* value of <0.05 was considered statistically significant. Prespecified endpoints were examined using two-tailed hypothesis testing. Exploratory endpoints were examined using one-tailed testing. Statistics were performed using SPSS (version 16.0; SPSS Inc., Chicago, IL, USA).

## Results

A total of 126 patients were approached for consent between June 2013 and April 2014. A total of 50 patients were included in the study. One patient was later excluded from analysis due to a discrepancy on the qualifying echocardiogram. Ten patients were randomized to receive placebo, 19 patients received 100 mg of niacin, and 20 patients received 500 mg of niacin. All enrolled patients completed the study. Baseline characteristics of each group are described in Table 1. The mean age was 68 ± 16, and 25 were men (51 %). None had known pulmonary vascular disease. Six patients (12.2 %) had a history of chronic obstructive pulmonary disease. Baseline TR jet velocity was 2.97 ± 0.38 m/s, and mean estimated pulmonary artery systolic pressure was 45.9 ± 9.5 mm Hg. There were no significant differences in baseline variables between study groups.

**Table 1:** Baseline characteristics in patients assigned to placebo, niacin 100 mg, and niacin 500 mg

	Placebo (*n* = 10)	Niacin 100 mg (*n* = 19)	Niacin 500 mg (*n* = 20)
Age (years)	69 ± 18.9	69 ± 15.5	67 ± 16.6
Male (N, %)	4 (40)	12 (63)	9 (45)
Hypertension (N, %)	6 (60)	12 (63)	16 (80)
Diabetes mellitus (N, %)	3 (30)	5 (26)	4 (20)
Hyperlipidemia (N, %)	1 (10)	3 (16)	4 (20)
COPD (N, %)	1 (10)	3 (16)	2 (10)
Smoker (N, %)	2 (20)	5 (26)	9 (45)
Left ventricular ejection fraction	56 ± 11.7 %	51 ± 13.3 %	56 ± 5.8 %

*COPD* chronic obstructive pulmonary disease

**Fig. 1:**
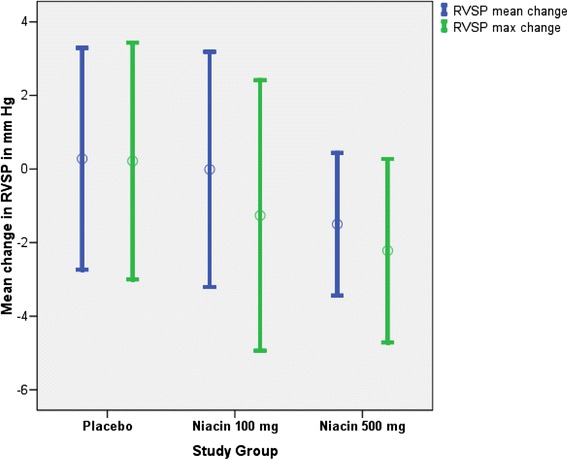
Mean and maximum change in RVSP in mm Hg in all study groups. Mean change in estimated right ventricular systolic pressure (RVSP) in mm Hg for placebo, niacin 100 mg, and niacin 500 mg measured 1 hour after medication administration. Data displayed are for the changes observed in average RVSP (*blue*), and in maximum RVSP (*green*) observed in up to ten consecutive Doppler measurements

The baseline mean estimated RVSP for all 49 subjects (25 male) was 51.9 ± 12.1 m/s. The primary endpoint of mean change in TR jet velocity was 0.016 ± 0.065 m/s in the placebo group, compared to −0.017 ± 0.065 m/s with niacin 100 mg, and −0.063 ± 0.038 m/s with niacin 500 mg (*P* = 0.63). The change in maximum estimated RVSP across the three drug groups was 0.2 ± 1.6 mm Hg, −1.3 ± 1.8 mm Hg and −2.2 ± 1.2 mm Hg (*P* = 0.62). In exploratory pairwise analysis in the high-dose niacin group (500 mg), the reduction in mean RVSP was from 50.9 ± 9.4 mm Hg to 48.7 ± 10.0 mm Hg (*P* = 0.09). Exploratory pairwise analysis in the other two groups yielded nonsignificant *P* values. There were no differences observed within strata of baseline RVSP (above or below median RVSP) or for age (Fig. 1).

## Discussion

We hypothesized that niacin-mediated prostaglandin release might lead to measurable acute effects on right ventricular systolic pressure in patients with pulmonary hypertension. In this pilot study, we have shown that major changes in RVSP were not observed after single-dose provocation with 100 mg or 500 mg of niacin. However, with a dose–response difference, our data are consistent with a possible small reduction in RVSP at the 500 mg dose. These data provide insights for future studies on the topic, in particular dosing, effects of dosing at 1 hour, and data for sample size determinations.

Long known is niacin’s principle side effect of vasodilatory flushing, exhibited after oral ingestion of large doses or immediate-release niacin. The physiological mechanism of flushing relates to an abnormally large concentration of prostaglandins released upon niacin absorption, predominantly PGD_2_ [7]. Ingested niacin binds to G protein-coupled receptor 109A (GPR109A), triggering a signal cascade leading to PGD_2_ formation and release [12]. Subsequent vasodilation only occurs after PGD_2_ binds to its receptor DP_1_, located on vascular muscle cells [8, 13]. This knowledge led to our hypothesis that pulmonary artery vasodilation may be a physiological correlate of niacin-induced prostaglandin release.

Niacin has not previously been investigated for its effects on pulmonary artery pressure. Indeed, new simple and low-cost treatments for PAH are needed. Low levels of vasodilating prostaglandins and high levels of vasoconstricting thromboxanes result in the high pulmonary pressures and resistance evident in patients with PAH [2, 4]. Long-term, intravenous administration of prostacyclin (PGI_2_) has seen the most success in treating this imbalance, providing symptomatic alleviation, hemodynamic improvement, and prolonged survival in patients [5, 6]. It has also been shown to significantly reduce echocardiographic measurements of maximum TR jet velocity after 12 weeks of therapy [14]. However, complications are common and new treatment options using synthetic prostacyclin analogues and other prostaglandins are continuously being explored. A particular limitation of such treatments is their expense.

Several opportunities exist for applying these results to future studies. First, without appropriate animal models or prior experience to guide us, we selected doses of 100 mg and 500 mg of niacin based largely on the maximal initial doses of immediate-release niacin that are tolerable. Based upon pharmacokinetic bioavailability, we estimated that 1 hour post administration would be the time of exposure to maximal PGD_2_ levels correlating to the typical period of cutaneous flushing. This study explored niacin’s (putatively via PGD_2_) ability to quantitatively reduce pulmonary arterial pressure assessed using TR jet velocity. Although we found no overall effect, several potential opportunities for future research emerged. First, our data suggests a positive trend with larger reductions of pulmonary arterial pressure at the highest dose (500 mg) of niacin. Second, our data are compatible with a small reduction in RVSP at the 500 mg dose, although we caution that this is a subgroup endpoint and found within exploratory statistical methods that require further hypothesis generation and testing. Our study supports the feasibility and safety of administration of niacin as a pulmonary vasodilator among patients with overall mild to moderate pulmonary hypertension. Given the indirect nature of assessment of RVSP using TR jet velocity, future studies should utilize more direct techniques such as pulmonary artery catheterization that, although invasive, would be more definitive and aid in the determination of the time course of effect. For example, intravenous prostacyclin in acute administration results in little change in pulmonary artery systolic pressure due to an offsetting effect of increased cardiac output in the setting of pulmonary artery vasodilation [14]. As niacin may stimulate a number of offsetting hemodynamic variables such as increased cardiac output in a setting of reduced pulmonary vascular resistance yielding no net effect on RVSP, the hypothesis that niacin induces pulmonary artery vasodilation may not be inconsistent with our initial findings.

### Limitations

We utilized TR jet velocity as a surrogate to pulmonary pressure given its noninvasive nature and the pilot study approach. Although accurate, the pressures derived are dependent on a number of assumptions and technical optimization. Although we attempted to eliminate interest variability through consistency in the sonographer and insonation views, a more direct approach utilizing pulmonary artery catheterization would be advised for more definitive evaluation. There are many different etiologies underlying PAH. Additionally, not all forms of PAH will respond equally to vasodilators. This introduces the possibility of a heterogeneous study population. We selected a niacin dose for this study based upon tolerance for initial dosing of the drug. If potential favorable effects on pulmonary artery pressure are shown through future studies, dose optimization during titration and sustained administration would be necessary. Our study was designed to detect a small change in TR jet velocity, although the standard deviation was larger than anticipated. Our data should aid future investigators in sample size determinations. Lastly, we empirically elected to assess the change in TR jet velocity after 60 minutes, based upon the time course of clinical flushing caused by increased circulating levels of PGD_2_. However, absorption rates between patients may vary, and indeed patients differed in the time when maximum flushing occurred, most prior to the second echocardiogram and a few after.

## Conclusions

Niacin, via stimulation of PGD_2_ release, may favorably reduce pulmonary artery pressure in patients with mild to moderate pulmonary artery hypertension. However, this pilot study did not meet its primary and secondary endpoints of change in mean and maximum TR jet velocity, while demonstrating a trend for dose response and possible reduction of RVSP at the 500 mg dose. Future studies utilizing direct measurements of pulmonary artery pressure and repeat niacin dosing are warranted.

## Additional files

Additional file 1:
**CONSORT flow diagram for the Niacin Study.** (DOCX 32 kb) Additional file 2:
**CONSORT 2010 Checklist for the Niacin Study.** (DOCX 38 kb)

## Abbreviations

ANOVA: analysis of variance
ALT: alanine transaminase
AST: aspartate transaminase
Bi-PAP: bi-level positive airway pressure
GPR109A: G-protein coupled receptor 109A
IVC: inferior vena cava
MGUH: MedStar Georgetown University Hospital
m/s: meters/second
NPO: nothing by mouth
NSAID: nonsteroidal anti-inflammatory drug
PAH: pulmonary arterial hypertension
PGD_2_: prostaglandin D_2_
PGI_2_: prostacyclin or prostaglandin I_2_
RVSP: right ventricular systolic pressure
TR: tricuspid regurgitation
