# Supplementary material for: A randomized pilot study on the effect of niacin on pulmonary arterial pressure
Source: Trials. 2015 Nov 21;16:530. doi: 10.1186/s13063-015-1013-6 (PMC4654874; doi:10.1186/s13063-015-1013-6)
Supplement: Additional file 1: — CONSORT flow diagram for the Niacin Study. (DOCX 32 kb) [file 13063_2015_1013_MOESM1_ESM.docx]

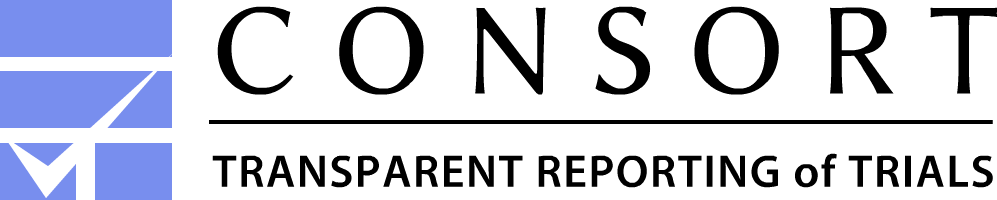


**A Pilot Study on the Effect of Niacin on Pulmonary Arterial Pressure**

## Analysis

## Enrollment

## Allocation

Randomized (n=50)

Assessed for eligibility (n=126)

Excluded (n=76 )

♦  Declined to participate (n=76)

Allocated to Placebo group (n=10)

♦ Received allocated intervention (n=10)

Allocated to Niacin dosing groups (n= 40).

♦ Received 100 mg Niacin (n= 19).

♦ Did not receive allocated intervention due to discrepancy on qualifying echocardiogram (n=1).

♦ Received 500 mg Niacin (n= 20).

Analysed (n=39)

Analysed (n=10)
